# How Can National Antimicrobial Stewardship Interventions in Primary Care Be Improved? A Stakeholder Consultation

**DOI:** 10.3390/antibiotics8040207

**Published:** 2019-10-31

**Authors:** Aleksandra J. Borek, Marta Wanat, Anna Sallis, Diane Ashiru-Oredope, Lou Atkins, Elizabeth Beech, Susan Hopkins, Leah Jones, Cliodna McNulty, Karen Shaw, Esther Taborn, Christopher Butler, Tim Chadborn, Sarah Tonkin-Crine

**Affiliations:** 1 Nuffield Department of Primary Care Health Sciences, University of Oxford, Radcliffe Observatory Quarter, Oxford OX2 6GG, UK; marta.wanat@phc.ox.ac.uk (M.W.); christopher.butler@phc.ox.ac.uk (C.B.); sarah.tonkin-crine@phc.ox.ac.uk (S.T.-C.); 2 Public Health England Behavioural Insights, London SE1 8UG, UK; Anna.Sallis@phe.gov.uk (A.S.); Tim.Chadborn@phe.gov.uk (T.C.); 3 Public Health England, London SE1 8UG, UK; Diane.Ashiru-Oredope@phe.gov.uk (D.A.-O.); Susan.Hopkins@phe.gov.uk (S.H.); Leah.Jones@phe.gov.uk (L.J.); Cliodna.McNulty@phe.gov.uk (C.M.); k.shaw7@nhs.net (K.S.); 4 Centre for Behaviour Change, University College London, London WC1E 6BT, UK; l.atkins@ucl.ac.uk; 5 NHS England and NHS Improvement, London SE1 6LH, UK; elizabeth.beech@nhs.net (E.B.); esther.taborn@nhs.net (E.T.); 6 NIHR Health Protection Research Unit in Healthcare Associated Infections and Antimicrobial Resistance, University of Oxford in Partnership with Public Health England, Wellington Square, Oxford OX1 2JD, UK; 7 University College London Hospitals, London NW1 2PG, UK; 8 NHS East Kent Clinical Commissioning Groups, Canterbury CT1 1YW, UK

**Keywords:** antimicrobial stewardship, antibiotic prescribing, primary care, implementation, behavior change, stakeholder consultation

## Abstract

Many antimicrobial stewardship (AMS) interventions have been implemented in England, facilitating decreases in antibiotic prescribing. Nevertheless, there is substantial variation in antibiotic prescribing across England and some healthcare organizations remain high prescribers of antibiotics. This study aimed to identify ways to improve AMS interventions to further optimize antibiotic prescribing in primary care in England. Stakeholders representing different primary care settings were invited to, and 15 participated in, a focus group or telephone interview to identify ways to improve existing AMS interventions. Forty-five intervention suggestions were generated and 31 were prioritized for inclusion in an online survey. Fifteen stakeholders completed the survey appraising each proposed intervention using the pre-defined APEASE (i.e., Affordability, Practicability, Effectiveness, Acceptability, Safety, and Equity) criteria. The highest-rated nine interventions were prioritized as most promising and feasible, including: quality improvement, multidisciplinary peer learning, appointing AMS leads, auditing individual-level prescribing, developing tools for prescribing audits, improving inductions for new prescribers, ensuring consistent local approaches to antibiotic prescribing, providing online AMS training to all patient-facing staff, and increasing staff time available for AMS work with standardizing AMS-related roles. These prioritized interventions could be incorporated into existing national interventions or developed as stand-alone interventions to help further optimize antibiotic prescribing in primary care in England.

## 1. Introduction

Conserving antibiotics by optimizing antibiotic prescribing to reduce the spread of antimicrobial resistance is a key public health priority both globally and nationally in the UK [1,2,3]. In England, 81% of antibiotics were prescribed in primary care in 2017 [4], and up to 23% of these are estimated to be prescribed inappropriately, mostly (unnecessarily) for self-limiting respiratory tract infections (RTIs) [5]. While antibiotic prescribing in primary care in England reduced by 13.2% between 2013 and 2017 [4], antibiotic use in the community is still higher than in several other European countries [6]. There is also a considerable variation in antibiotic prescribing between general practices, with many practices remaining high prescribers [7], and between practices and other types of healthcare providers in the community (e.g., out-of-hours, urgent care) [4]. The variations in antibiotic use are not (fully) explained by differences in patient characteristics, such as clinical presentation or prevalence of comorbidities [7,8].

Changing healthcare professionals’ (HCP) prescribing behaviors can help reduce antibiotic use and many factors influencing antibiotic prescribing for RTIs in primary care have been identified [9,10,11,12,13]. A range of antimicrobial stewardship (AMS) interventions targeting HCPs have been developed, with many shown effective in trials [14,15,16]. However, despite the recent decrease in antibiotic prescribing and availability of AMS interventions, further optimizing and reducing inappropriate antibiotic use in English primary care remains critical, especially among the higher prescribers. Further progress has been included in the recent National Health Service (NHS) long-term plan [17] and is required to meet the UK five-year target to reduce antibiotic prescribing in community by 25% by 2024 [2]. Behavioral science evidence shows that to be effective, behavior change interventions need to target relevant determinants of behavior and needs of the targeted population, and fit within the contexts where they are implemented [18,19]. Thus, further improving antibiotic prescribing might involve adapting and implementing effective AMS interventions that have not been yet widely used in England [14], and/or addressing contextual and implementation-specific influences experienced by those using AMS interventions [11].

A recent study aimed to explore nationally implemented AMS interventions in the UK and the extent to which they target behaviors related to antibiotic use. Twenty-two interventions for primary care prescribers and eight for community pharmacy staff were identified, targeting on average 5.8 HCPs’ behaviors [10]. A follow-up study identified barriers and facilitators to appropriate antibiotic prescribing in primary care and found nine interventions evaluated in the UK and shown effective at reducing antibiotic prescribing [11]; these included five research-only interventions [20,21,22,23,24] and four nationally available interventions: communication skills training [25], FeverPAIN clinical score [26], the TARGET toolkit [27], and the Chief Medical Officer’s letters with prescribing feedback to the highest-prescribing practices [28]. Analyzing the behavioral content of the identified AMS interventions and comparing the extent to which they address relevant behaviors and key barriers and facilitators led to identification of potential changes to, or gaps to be addressed by, AMS interventions. However, such theoretical analysis lacks the insight from the targeted population and intervention users, and does not address factors related to context and implementation of interventions. Therefore, we aimed to build on this recent research by consulting stakeholders (i.e., HCPs from general practices, out-of-hours, community pharmacies and commissioning organizations in England) to: (a) identify barriers and facilitators to optimizing antibiotic prescribing and implementing AMS interventions specific to their settings, (b) generate suggestions for improvements of AMS interventions in their specific, primary care settings in England, and (c) prioritize interventions (using pre-specified feasibility and acceptability criteria). This paper reports the findings of this stakeholder consultation.

## 2. Results

### 2.1. Stakeholder Focus Group and Telephone Interviews

Twelve stakeholders attended the focus group and three participated individually by telephone. Seven were representatives from Clinical Commissioning Groups (CCGs, i.e., organizations responsible for planning and commissioning of health care services for their local areas in England), three were from NHS England, two from out-of-hours (OOH) organizations, one from a chain of community pharmacies, and two were general practitioners (GPs).

In the first part, the stakeholders discussed barriers and facilitators to optimizing antibiotic prescribing in primary care settings. These are summarized in Table 1 and reported in more detail in Supplementary Materials (Boxes S1–S4). In brief, as key facilitators to optimizing antibiotic prescribing, the stakeholders reported the availability of many AMS interventions, and awareness of healthcare professionals of the need for appropriate and prudent antibiotic prescribing. As one of the key challenges they reported a variation in use (and sometimes low uptake) of interventions between organizations and HCPs. This was exacerbated by barriers including: limited dissemination of information about specific interventions; insufficient time to engage with interventions (related to large workloads and multiple competing priorities); lack of clarity on which interventions to engage with (influenced by a perceived large number of interventions); insufficient initiatives with professionals collaborating across networks (e.g., involving GPs, pharmacists, nurses) which fueled perceptions of ‘working in silos’.

In the second part of the consultation, the stakeholders identified challenges to implementing specific current AMS interventions and made suggestions for improvements. These suggestions were compiled, separately for each primary care setting, and are summarized in Table 1 (and reported in more detail in Supplementary Materials, Boxes S1–S4). Key suggestions included: offering financial incentives; mandating certain target behaviors (e.g., making AMS training a mandatory part of professional development or appraisal); regularly auditing prescribing in all practices and of individual prescribers and, based on this, providing interventions tailored to local contexts and individual needs and addressing specific reasons for suboptimal prescribing; developing multi-professional networks and learning groups to promote communication, collaboration and learning between different professions (e.g., GPs, nurses, pharmacists); incorporating interventions nationally within existing clinical systems; and using point-of-care (POC) diagnostics, such as C-Reactive Protein (CRP) tests or throat swabs (although stakeholders expressed ambivalent views on these). No suggestions were identified for walk-in/urgent care centers as no stakeholders were from this specific setting. However, it was agreed that some of the suggested interventions may be relevant to this setting. 

### 2.2. Revising and Selecting Intervention Suggestions

Additional intervention components were identified based on available evidence on AMS interventions shown to be effective in the UK [11]. In addition, steering group members and the research team provided additional suggestions based on their experience and knowledge of current national AMS policy. These were added to the list of suggestions made by the stakeholders. Altogether, 45 intervention suggestions were identified. Some involved modifications of existing interventions or their implementation (e.g., relating to dissemination of information), whereas others involved new intervention components (e.g., that could form a part of an existing intervention or be implemented as a stand-alone intervention).

In order to identify which influences on antibiotic-related prescribing behaviors the suggested interventions aimed to address, the interventions were mapped onto barriers and facilitators to appropriate antibiotic prescribing. These influences were identified in the literature review [11] and by the stakeholders. After revising and selecting intervention suggestions, 31 interventions were included in an online survey comprising: seven suggestions potentially applicable to all settings, ten suggestions specifically for general practice, nine for OOH, and five for community pharmacy. The full list of the 45 identified interventions is available in Supplementary Materials (Table S1), together with barriers and facilitators that they addressed, source of how each intervention was identified, and indicating which interventions were included in or excluded (with reasons) from the survey.

### 2.3. Stakeholder Survey and Prioritized Intervention Suggestions

Out of 40 stakeholders invited to complete the survey, 15 (38%) completed it. Seven respondents indicated that they worked (or had expertise) in general practice, five in CCGs, four in OOH, three in walk-in/urgent care centers, one in community pharmacy, and four in other settings (i.e., two working across settings; one in community hospital; one in e-learning for healthcare professionals). The respondents reported between 4 and 23 (mean 10.7) years of relevant experience. The APEASE scores for each intervention and setting are reported in full in Supplementary Materials (Tables S2–S5).

Nine unique interventions were prioritized (Table 2). Three interventions were prioritized for OOH and community pharmacy, and four were prioritized for general practice and walk-in/urgent care centers (as two of the highest-scoring interventions for these two settings had even scores). As some interventions were assessed for multiple settings, four interventions were prioritized for multiple settings: ‘(2) Multi-disciplinary small group learning’ was prioritized for general practice, walk-in/urgent care centers, and community pharmacy; ‘(3) Appointing AMS leaders’ for general practice and OOH; ‘(7) Agreeing on a consistent local approach to antibiotics’ for walk-in/urgent care centers and community pharmacy; ‘(8) Providing online AMS training to all patient-facing staff’ for walk-in/urgent care centers and community pharmacy.

The lowest scoring intervention (with 22.7% of the maximum APEASE score) was ‘providing diagnostic point-of-care CRP testing, including training in using it, interpreting the results and maintaining the equipment’ in community pharmacy setting, which was assessed by only five out of 11 respondents as relevant to the setting, by two as practical and acceptable and by none as affordable. This intervention was also rated as the second lowest for general practice (44% of the maximum APEASE score), walk-in/urgent care centers (48.7%) and OOH (50%). Participants’ comments provided as free text in the survey indicated that cost and funding, time to do the tests, and concern about over-use of the tests by patients and clinicians were considered the main barriers to using this intervention; for example:

“*Would need to have clear guidance and uptake would depend on who was funding [POC CRP tests]. Barriers to GP practices are cost of equipment and cost of tests, as well as time it takes to perform the test when only have 5–10 min consultation and test takes a few minutes to perform so practical and affordability issues are the main barriers*.”

“Concerns [that POC CRP tests] may increase attendance to ‘get a test’. May involve clinicians overly relying on a test which is not always accurate or there may be a time lag in the increase in CRP. Time taken in consultation to administer test is a barrier and test strips are costly.”

The two second lowest scoring (with 33.3% of maximum APEASE score) interventions were also in community pharmacy setting; although both were assessed by only three participants. One was ‘providing training and resources to structure the way(s) of asking patients the right questions about self-limiting infections and identifying red flags to help decide what to advise patients’. In comments, the participants suggested that: “*this sort of training is available* via *CPPE, however uptake is voluntary*” and “*this should already be done as part of the core community pharmacy contract*.” The other intervention was ‘promoting the use of patient records by pharmacists (e.g., by digital prompts) to review whether antibiotics were prescribed appropriately’. Participants’ comments suggested that:

“*At present community pharmacists do not have access to enough information to be able to do this effectively. There may need to be specialist clinical training for community pharmacists to do this*.”

“*The relevance will depend on where the community pharmacist is in the patient pathway. If contractual levers remain as is the community pharmacist may require remuneration*.”

Other lowest scoring interventions (see a full list in Supplementary Materials) were: ‘providing information on opening hours of all local healthcare services’ for general practice (31.8% of maximum APEASE score) which was scored particularly low on ‘effectiveness’, and ‘co-organizing national AMS events together with different professional networks’ for OOH and walk-in/urgent care centers (50% and 38.5%, respectively) which was rated low on ‘affordability’.

## 3. Discussion

The stakeholder consultation identified setting-specific barriers and facilitators to current antibiotic optimization, and generated and prioritized suggestions for improvements of AMS interventions in primary care in England. Stakeholders’ appraisal of relevance, feasibility and acceptability of 31 intervention suggestions led to nine interventions being prioritized across settings. These prioritized interventions address some of the identified influences on antibiotic prescribing. They could be incorporated as part of existing AMS interventions or further developed and implemented as stand-alone interventions.

### 3.1. Implications within the Context of Current AMS Research and Practice

The interventions assessed and prioritized (or not) by the stakeholders build on AMS interventions currently implemented in England [10] and effective interventions tested in UK-based research studies [11]. How the 31 interventions fit with current research and practice, and how those prioritized may be implemented, is discussed below and summarized in Table 3. Interventions are grouped by intervention ‘type’, for ease of reference.

*Improving engagement with AMS training and resources:* There are many AMS interventions available, including AMS training and resources tested and shown effective in trials [21,25,27]. For example, the TARGET antibiotic toolkit (with training and resources primarily targeted at GPs) is available online [29] and practice workshops promoting the TARGET resources were shown effective [27]. Similarly, the STAR online communication skills training (with a practice seminar) was shown effective in a trial [25] and is now available online on the clinical professional development website [30]. CCG professionals report that HCPs are aware of, and promote or engage with, different AMS training and resources (such as the TARGET toolkit), but time, reaching the correct people and lack of clarity on which online training to promote were reported as the main issues with AMS education [31]. Similarly, the stakeholders in our study identified improving engagement with AMS interventions as a challenge, influenced by lack of time and priority, clarity on which interventions to use, incentives, and opportunities (e.g., protected time, training for HCPs in organizations other than general practice). Current AMS interventions may, at least in theory and research trials, facilitate change but improving the uptake of and engagement with AMS interventions in the real world is critical to further optimizing antibiotic use. This may be facilitated by the ‘train the trainers’ opportunities provided by the TARGET team [29] and by increasing numbers of pharmacists appointed in primary care settings with AMS as part of their roles. The importance of improving engagement with AMS training and resources was reflected by suggestions prioritized by the stakeholders, such as organizing ‘(2) multi-disciplinary small group learning’ (that could be delivered face-to-face, in addition to online resources, and focus on identifying challenges and solutions specific to local contexts), ‘(8) providing online AMS training to all patient-facing staff’ (rather than, as currently, targeting primarily GPs), and ‘(9) increasing staff time available for AMS work and standardizing AMS roles’. Another suggestion to improve engagement with AMS training that was made by the stakeholders but received medium APEASE score was to make the AMS training mandatory in general practices.

*Enhancing prescribing data monitoring and feedback:* Antibiotic prescribing data has been publicly available and fed back to prescribers by a vast majority of CCGs for many years and interventions involving prescribing feedback have been shown effective [24,28]. In contrast, detailed action planning is rarely used (reported for only 16% of CCGs [31], often due to insufficient time for it [32]), as is feedback on individual prescribing—both of these strategies were prioritized. Including them may enhance the impact of monitoring of and feedback on prescribing by specifying and tailoring actions (setting goals and/or ‘if-then’ plans) to address specific reasons for inappropriate antibiotic prescribing and by activating individual accountability for prescribing. Based on available evidence from lifestyle-related interventions, such behavioral regulation strategies (i.e., self-monitoring, especially when combined with other ‘regulatory’ techniques such as goal-setting, problem solving or ‘if-then’ plans) and individual tailoring can be effective behavior change techniques (e.g., [33,34]). Moreover, while monitoring/auditing of, and feedback on, prescribing have likely contributed to reduced antibiotic prescribing in general practice, the stakeholders reported barriers to using these strategies in OOH, such as lack of stable patient population and different computer systems. Developing tools to enable and automate prescribing audit and provision of individualized feedback might further optimize antibiotic prescribing in OOH.

*Ensuring consistency in AMS approaches:* The suggestion to ‘(7) agree on a consistent local approach to antibiotics’ (prioritized for walk-in/urgent care centers and community pharmacies and also highly scored for general practice and OOH) highlights the importance of consistency in managing infections and reinforcing consistent messages to patients by different HCPs and across organizations. It could be implemented by developing and agreeing within-and between-organization action plans or protocols, for example, on using patient leaflets and other resources promoting messages about infection prevention and self-care. The importance of consistency between HCPs in antibiotic prescribing and the messages given to patients was also reflected in the following interventions prioritized by the stakeholders: ‘(2) multidisciplinary small group learning’ and ‘(8) providing online AMS training to all patient-facing staff’. Moreover, four interventions were prioritized for multiple settings (i.e., ‘(7) agreeing on a consistent local approach to antibiotics’; ‘(2) multi-disciplinary small group learning’; ‘(8) providing online AMS training to all patient-facing staff’; ‘(3) appointing AMS leaders’) and could be considered for implementation across settings and by involving HCPs from different professional networks. This could help promote a more integrated, system-wide approach to AMS [17]. Respondents in a recent survey representing 99% of Clinical Commissioning Groups (CCGs) reported that AMS training was targeted primarily at GPs, compared to 67% of CCG professionals reporting focus on other prescribers, 42% on all practice staff and only 28% on OOH staff; consequently, a system and practice-wide approach was one of the top suggestions for AMS training by the CCG professionals [31].

Finally, it may also be important to consider interventions that have not been prioritized by stakeholders. Providing POC CRP tests was among the lowest-scoring suggestions in all settings. The stakeholders considered it not affordable or practical to deliver. While POC CRP testing is supported by examples of countries with low prescribing rates that routinely use it (e.g., Netherlands) and trial evidence showing it as an effective and safe strategy to reduce antibiotic prescribing for RTIs in general practice [14,35], it may not have sustained effects on prescribing behavior [36] and is often met with mixed views on its usefulness and feasibility from HCPs [37,38]. Moreover, ‘co-organizing national AMS events for participants from different professional networks to facilitate multi-disciplinary AMS work’ and ‘promoting the use of patient records by pharmacists to review whether antibiotics were prescribed appropriately’ seemed to be considered by stakeholders as unaffordable; ‘providing information on opening hours of local healthcare services’ (to reduce prescribing when concerned about limited access to healthcare) was considered unlikely to be effective; and ‘providing training and resources to structure the ways of asking patients the right questions about self-limiting infections and identifying red flags to help decide what to advise patients’ in community pharmacy was seen as of low relevance. Our findings suggest that these interventions might be less promising ways to optimize antibiotic use for RTIs, at least as seen by the small number of stakeholders consulted in this study. Further research may need to explore and identify ways to address barriers related to these interventions with a larger group of stakeholders and intervention users. In addition to the nine prioritized interventions and five lowest-scoring interventions, there were also 17 other interventions included in the survey and another 14 suggestions that were not prioritized for inclusion in the survey (all available in the Supplementary Materials) that could potentially be considered and refined.

### 3.2. Strengths and Limitations

This study was based on expert input from stakeholders and the study steering group, who had practical experience and knowledge about national AMS interventions and policy. Views of stakeholders and intervention users are critical, yet at times under-represented, in theoretical approaches to developing and refining AMS interventions. Our study, focused on views of commissioners and other key stakeholders, may help address this gap and provide some indication for future research on implementation of AMS interventions. However, our findings need to be interpreted with caution. The stakeholders were identified by the steering group members from their own professional networks, and out of 40 stakeholders invited to the focus group and survey, only 15 responded. It is possible that a larger number of stakeholders or intervention users might have generated suggestions for, and prioritized, different interventions. The stakeholders had varied levels of experience and knowledge of AMS interventions and different settings (with less experience specific to OOH, walk-in centers and community pharmacies). This made it difficult to identify very specific improvements to current AMS interventions and to capture a wider range of views from different organizations and settings. As the study focused on stakeholders and interventions in England, the generalizability of the findings may be limited beyond England.

## 4. Materials and Methods

The following steps were taken: (1) a stakeholder focus group and telephone interviews to identify barriers and facilitators to appropriate antibiotic prescribing and to generate intervention suggestions; (2) revision and selection of intervention suggestions; (3) a stakeholder survey to assess and prioritize interventions according to pre-specified criteria of relevance, feasibility and acceptability. We focused on interventions targeting HCPs’ antibiotic prescribing for RTIs in the following settings: general practice, out-of-hours (OOH), walk-in/urgent care center, and community pharmacy. The study was reviewed by the University of Oxford Clinical Trials and Research Governance team and classified as service development, and as such it did not require research ethics review. Participants were free to participate in any stage of the consultation and withdraw at any point without any consequences. They were offered reimbursement of travel expenses but no payment for participation.

### 4.1. Stakeholder Focus Group and Telephone Interviews

Relevant stakeholders (including HCPs with expertise in and/or experience of antibiotic prescribing in relevant settings) were identified by the study steering group members. The steering group included experts in AMS with expertise in, and experience of, designing and implementing AMS interventions and influencing national AMS policy in the UK. Identified stakeholders were invited by email to attend a 3-hour face-to-face focus group held at Public Health England premises in London. They were followed up once in cases of non-response. Those who could not attend the focus group in person were invited to a telephone interview.

The aims of the focus group were (a) to identify barriers and facilitators to appropriate antibiotic prescribing and to implementing AMS interventions in relevant settings; and (b) to generate suggestions for improvements of current AMS interventions and/or their implementation or for new interventions addressing the identified barriers and facilitators. In the first part of the focus group, the stakeholders were presented with barriers and facilitators identified from a literature review [11]. This was followed by a discussion of stakeholders’ experiences and examples of these, and of any other influences on antibiotic prescribing (especially in settings under-represented in the literature, such as community pharmacy, OOH and walk-in centers). In the second part of the focus group, the stakeholders were presented with examples of nationally available AMS interventions and/or interventions trialed in research studies in the UK. This was followed by a discussion about stakeholders’ experiences of using these interventions, any challenges to their use or implementation, and suggestions for improvements and/or any new interventions.

The focus group discussions were facilitated by two researchers in a semi-structured way, which included short presentations and general discussion, followed by more specific questions about participants’ views, experiences and examples. A third researcher took detailed notes. The focus group was audio-recorded and the recording was subsequently used to check the notes and add more detail. Additionally, participants were provided with handouts including the information on barriers and facilitators and AMS interventions identified in the literature, and with questions for discussion. They could provide additional comments on the handouts and return them to the researchers. The stakeholders interviewed by telephone were given information and asked questions in a similar format to the focus group. Detailed notes were made on each telephone interview. All suggestions made during the focus group, telephone interviews and on the handouts were collated and summarized. The summary of notes was used to generate a list of barriers and facilitators and a list of intervention suggestions.

### 4.2. Revision and Selection of Intervention Suggestions for a Survey

Barriers and facilitators to appropriate antibiotic prescribing and effective research interventions were identified in a literature review [11]); these were used to generate additional suggestions which were added to the list of interventions identified by the stakeholders. The intervention suggestions were divided into those potentially applicable to all settings or specific to each relevant setting. They were mapped onto barriers and facilitators identified by the stakeholders and in a preceding literature review [11].

The list of suggested interventions was reviewed by the researchers (AB, MW, STC, AS) and steering group members (remaining authors) who provided feedback on and suggestions for rephrasing the suggested interventions, and made additional suggestions that were added to the list of interventions. To reduce a relatively large number of identified suggestions, the comments from the steering group members (who have knowledge of existing AMS interventions and those currently in development) were used to prioritize selection of interventions for the survey; suggestions were excluded if they were considered unfeasible, already implemented, already under development, or insufficiently specific.

### 4.3. Stakeholder Survey and Prioritization of Interventions

An online survey was used for the stakeholders to assess intervention suggestions using the APEASE criteria [39]: Affordability (is an intervention affordable?), Practicability (can it be delivered easily?), Effectiveness (is it likely to be effective?), Acceptability (is it acceptable to staff?), Side effects and safety (is it safe to implement?), and Equity (can it avoid inequalities in patient care?). The survey was designed and delivered using online Survey Monkey software (www.surveymonkey.com). Stakeholders identified by the steering group (those invited to the focus group) were invited by email, including a brief description about the survey and a link to complete it online. Participants were sent one reminder; responses were anonymous.

The survey asked about participants’ roles, work setting or expertise, and years of relevant experience. Participants were then presented with interventions that could be potentially applicable to all settings (i.e., general practice, OOH, walk-in/urgent care center, community pharmacy) and suggestions specific to each setting: general practice, OOH, and community pharmacy. For each suggestion, participants were asked to: (a) assess whether the intervention was relevant to the setting, and, if yes, then (b) to assess it, for that setting, using the APEASE criteria.

Participants could skip questions and whole sections so the numbers of respondents that assessed each intervention for each setting varied. We calculated the number of responses for each intervention in each setting, the maximum possible APEASE score for each intervention/setting, and the actual APEASE score for each intervention/setting. The percentage of the maximum possible APEASE score obtained for each intervention/setting was calculated to allow comparison between interventions.

In order to identify interventions which were rated highly, the following criteria were used to prioritize interventions in each setting: (a) at least 50% of respondents for that question assessed the intervention as relevant to that setting; and (b) intervention was scored as one of the top three (or four in cases of equal scores) based on the percentage of the maximum APEASE score.

## 5. Conclusions

The study identified a number of barriers and facilitators to optimizing antibiotics and engaging with AMS interventions, and possible ways in which current AMS interventions in primary care in England could be optimized. Nine interventions were prioritized by stakeholders as relevant, acceptable, affordable and feasible to implement in English primary care. They involve suggestions to help improve engagement with existing AMS training and resources (e.g., by face-to-face small group learning, tailoring AMS training to all patient-facing staff rather than prescribers only), enhancing data monitoring and feedback (e.g., audit and feedback on individual’s prescribing, or developing tools to automate prescribing audits), and promoting consistency in AMS approaches across healthcare professionals and services (e.g., by agreeing consistent local approaches, upskilling all patient-facing staff). These can be adapted and further developed as part of current AMS initiatives. Additionally, stakeholders prioritized suggestions to incorporate AMS interventions into clinical systems at a national level and enable sharing of patient information between general practice and OOH clinical systems. Future work needs to also focus more on addressing particular barriers to engagement with specific AMS interventions.

## Figures and Tables

**Table 1:** Summary findings from stakeholder focus group and interviews.

Examples of Identified Facilitators (F) and Barriers (B)	Examples of Suggestions for Intervention Improvements or New Interventions
Relevant to all settings	
F: Availability of many AMS interventions and guidelines. F: Consistency of AMS/antibiotic-related messages and advice across HCPs and organizations. F: Knowing practice and prescribers’ prescribing rates and resistance rates. B: Feeling of guideline ‘overload’ and lack of time to read them. B: Lack of clarity on which AMS interventions should be used; variation in use of interventions across HCPs and organizations. B: Insufficient time, high workloads, and related decision-making fatigue. B: Insufficient collaboration between professional networks and organizations.	Incentivizing or mandating engagement with AMS training and other interventions. Making tools/interventions easy to use by incorporating them into clinical systems. Making professional networks more multi-professional and promoting multi-professional collaborations and learning. Providing better/easier access to data on prescribing data linked with resistance data. Addressing primary care HCPs’ concerns about sepsis.
Relevant to general practice	
B: Prescribing antibiotics remaining to be seen as easier and quicker than not prescribing (especially under time pressure). B: Prescribing antibiotics ‘just in case’ prior to limited access to healthcare (e.g., before a weekend). B: Prescribers (e.g., locums) not using unique prescriber codes, making it difficult to audit prescribing.	Financial incentives for practices with antibiotic prescribing targets. POC CRP testing (but mixed views due to concerns about costs and unintended consequences). Auditing prescribing in all practices and by all prescribers, with feedback and tailored approaches to address specific issues. Peer review of prescribing in practices. Training patient-facing practice staff in signposting patients and self-care advice.
Relevant to out of hours (OOH)	
B: Lack of stable patient population. B: Prescribers not using unique prescriber codes. B: Lack of accountability for prescribing. B: Variation in awareness of local guidelines. B: Lack of/limited support from commissioners. B: Different clinical systems limiting access to patient records.	Developing tools/system to enable/ automate prescribing audits in OOH. Making AMS interventions (e.g., training) provided by commissioners available and improving dissemination of information about them to OOH staff. Improving induction of new prescribers in OOH to ensure awareness of local guidelines.
Relevant to community pharmacy	
B: Variation in skills and experience between pharmacy staff, with some having low confidence in providing self-care advice. B: Limited access to POC diagnostics across pharmacies and concern about using them for financial benefit. B: Different computer systems limiting access to, and use of, patient records.	Providing training in giving self-care advice to improve skills and confidence of staff. Providing access to POC diagnostics and training to help pharmacy staff distinguish between serious and less serious illness (thus improving confidence in giving self-care advice). Promoting use of patient records to identify potentially inappropriate use of antibiotics.

**Table 2:** Interventions prioritized by stakeholders.

Prioritized Interventions (Short Title with Detailed Description)	Setting(s) for Which Interventions Were Prioritized (% of Max. APEASE Score)	Facilitators (F)/Barriers (B) Addressed by Interventions
1. Standardized quality improvement with tailored advice and action planning Prescribing advisors or practice prescribing/AMS leads to carry out standardized quality improvement (e.g., supported by IT system functionality) and use prescribing data to identify underlying reasons for high/inappropriate antibiotic prescribing, provide tailored advice to prescribers and agree practice action plans (e.g., practice plan to reduce immediate antibiotic prescribing for acute cough).	General practice (84.9)	F: Advice from colleagues when uncertain or to reinforce appropriate prescribing decisions; perceptions of own prescribing compared to others.
2. Multi-disciplinary small group learning Multi-disciplinary small group learning (e.g., including local GPs, nurses, pharmacists, CCG staff) to identify ways to improve implementation of AMS initiatives and share local examples of good practice and actions taken by others as part of AMS.	General practice (84.5), Walk-in/urgent care centers (61.5), Community pharmacy (56.1)	F: Learning from peers on whether they can improve and how, and about alternative prescribing techniques.
3. Appointing AMS leaders Appoint AMS lead prescribers in all practices/OOH sites to lead on AMS-related issues, e.g., by organizing practice meetings about AMS, disseminating information about new guidelines, encouraging peers to implement interventions.	General practice (83.3), OOH (91.7)	B: Lack of a leader to lead on, and encourage engagement with, AMS-related issues.
4. Auditing individual prescribing Audit prescribing of individual prescribers in general practices, to be done by local prescribing advisors, practice prescribing/AMS leads or practice pharmacists, and provide individual feedback on prescribing, identify underlying reasons for high/inappropriate antibiotic prescribing, provide tailored advice and agree individual action plans (e.g., individual prescriber’s plan to reduce immediate antibiotic prescribing for acute cough).	General practice (83.3)	F: Having prescribing monitored and audited, receiving feedback on prescribing. B: Lack of accountability for prescribing.
5. Developing tools/system for auditing prescribing Develop tools/system to enable (automated) audit of prescribing in OOH and provision of personalized feedback and advice.	OOH (77.8)	B: Auditing prescribing in OOH impossible or difficult due to not being linked to population or area.
6. Improving inductions for new prescribers Improve induction for new prescribers in OOH to ensure knowledge of relevant local guidelines (e.g., indications for antibiotic prescribing, first-line antibiotics) and organization-agreed approaches to prescribing antibiotics.	OOH (77.8)	B: Lack of awareness/knowledge of local guidelines by new/locum GPs in OOH.
7. Agreeing on a consistent local approach to antibiotics Agree on a consistent local approach to antibiotic prescribing within an organization, such as a general practice, out-of-hours, walk-in center or community pharmacy, for example, by agreeing an AMS-related action plan, a practice protocol on treating certain infections and/or following national or local guidelines.	Walk-in/urgent care centers (65.4), Community pharmacy (59.1)	B: Inconsistent approaches to antibiotic prescribing. F: Adopting guidelines or evidence as a standard practice (with intention to follow them).
8. Providing online AMS training to all patient-facing staff Provide online AMS training to all patient-facing staff within an organization to improve (and minimize variation in) skills to ensure a consistent approach to providing advice to patients and antibiotic prescribing for respiratory tract infections.	Walk-in/urgent care centers (62.8), Community pharmacy (59.1)	B: Variation in the skills and experience among staff.
9. Increasing staff time for AMS work and standardizing AMS roles Increase staff time available to work on AMS (within relevant organizations) and standardize the AMS-related roles; for example, all organizations to have adequate number of prescribing advisors and/or pharmacists to work more closely with practices, OOH, walk-in centers and community pharmacies (e.g., by auditing prescribing, disseminating information, providing training and advice).	Walk-in/urgent care centers (61.5)	F: Advice from and influence of relevant experts.

**Table 3:** Proposed AMS interventions and how they fit with current research and practice.

Types of AMS Intervention	Effective Intervention Trialed in the UK? ^1^	Intervention Implemented Nationally? ^2^	Interventions Suggested and Prioritized by Stakeholders (Green—Prioritized Interventions, Indicated by Numbers, e.g., (1); Orange—Lowest Scoring, White—Mid Scoring or No Suggestions) ^3^
AMS training and resources	Yes [21,25,27]	Yes (e.g., TARGET [29], STAR [30])—but: online only, targeted mainly at prescribers, varied uptake/engagement	(2) Multi-disciplinary small group learning (8) Providing online AMS training to all patient-facing staff (9) Increasing staff time for AMS work and standardizing AMS roles
			- Online training promoting increased use of delayed/back-up antibiotic prescriptions - Making AMS training mandatory
Antibiotic prescribing data monitoring and feedback	Yes [24,28]	Yes—data publicly available but: varied provision of feedback; lack of national data/feedback on individual prescribing; varied use of prescriber codes	(1) Standardized quality improvement with tailored advice and action planning, (4) Auditing individual prescribing with tailored advice and action planning (5) Developing tools/system to enable (automated) audit of prescribing in OOH
			- Promoting/regulating use of unique prescriber codes to enable individual prescribing feedback - Improving dissemination of data on local antimicrobial resistance patterns - Encouraging GPs to peer review each other’s antibiotic prescribing
			- Making antibiotic prescribing/infection audit in OOH mandatory
Patient leaflets	Yes [21,22]	Yes—but in general practice and OOH only	Promoting routine interactive use of patient leaflets (in community pharmacy)
Clinical decision support tools	Yes [20,26]	Yes—but uptake varies	[No interventions/suggestions for improvements were identified.]
Agreeing a consistent approach to antibiotics	Yes [23]	No	(7) Agreeing on a consistent local approach to antibiotics, e.g., AMS-related action plan, protocol (2) Multi-disciplinary small group learning (8) Providing online AMS training to all patient-facing staff (so that they give consistent messages to patients)
			Co-organizing national AMS events with different professional networks
POC CRP testing	Yes [21]	No	Providing point-of-care CRP tests
Prescribing guidelines	No trial evidence for specific guidelines	Yes—but guidelines vary locally	(6) Improving inductions for new prescribers in OOH to ensure knowledge of local guidelines and organization-agreed approaches to prescribing antibiotics
AMS leadership	No trial evidence	Yes—but roles vary, little available time	(3) Appointing AMS leaders in all practices to lead on AMS-related issues (9) Increasing staff time for AMS work and standardizing AMS roles
			- Using respected and trusted, national and local experts to promote AMS
AMS campaigns	No trial evidence	Yes	[No interventions/suggestions for improvements were identified.]
Other interventions for general practice and OOH	No	No	- Incorporating interventions into clinical systems nationally - Making patient information and history available on OOH IT system, and OOH information on GP IT system to enable follow up
			- Providing information on opening hours of local healthcare services to prevent higher prescribing on Fridays
Other interventions for community pharmacy	No	No	- Pharmacy staff to prompt GPs to review long-term and repeat antibiotic prescriptions - Encourage pharmacists to feedback to GPs where antibiotics were not prescribed according to guidelines
			- Promote the use of patient records by pharmacists to review whether antibiotics were prescribed appropriately - Provide training and resources to structure the way(s) of asking patients the right questions about self-limiting infections and identifying red-flags to help decide what to advise patients

Notes: ^1^ Nine UK-based studies of effective AMS interventions [8,20,21,22,23,24,26,27,28] were identified and are reported elsewhere [11]. ^2^ Twenty six nationally implemented AMS interventions were identified previously and are reported elsewhere [10]. ^3^ The nine prioritized interventions are numbered as in Table 2 and include the highest-scoring interventions (3–4 per setting) (green rows). Lowest-scoring interventions (3 per setting) are in orange rows; the remaining interventions with the APEASE scores in the middle are in white rows. All APEASE scores for each intervention and setting are reported in the Supplementary Materials (Tables S2–S5).

## Supplementary Materials

The following Supplementary Materials and Data are available online at https://www.mdpi.com/2079-6382/8/4/207/s1: Box S1. Summary findings from stakeholder consultation: relevant to all settings. Box S2. Summary findings from stakeholder consultation: relevant to general practice. Box S3. Summary findings from stakeholder consultation: relevant to out-of-hours. Box S4. Summary findings from stakeholder consultation: relevant to community pharmacy. Table S1. All identified intervention suggestions. Table S2. Stakeholder ratings of interventions for general practice. Table S3. Stakeholder ratings of interventions for out-of-hours. Table S4. Stakeholder ratings of interventions for walk-in/urgent care centers. Table S5. Stakeholder ratings of interventions for community pharmacy.

## Abbreviations

AMS: Antimicrobial Stewardship; CCG: Clinical Commissioning Group; CRP: C-Reactive Protein; GP: General Practitioner; HCP: Healthcare professional; NHS: National Health Service; NIHR: National Institute of Health Research; OOH: Out-of-hours; POC: Point-of-care; RTI: Respiratory tract infection.

## References

[1] Davies S., Gibbens N. (2013). UK Five Year Antimicrobial Resistance Strategy 2013 to 2018.

[2] Department of Health and Social Care (2019). UK Five Year Action Plan for Antimicrobial Resistance 2019 to 2024.

[3] World Health Organization (2015). Global Action Plan to Control the Spread and Impact of Antimicrobial Resistance in Neisseria Gonorrhoeae.

[4] Public Health England (2018). English Surveillance Programme for Antimicrobial Utilisation and Resistance (ESPAUR). Report 2018.

[5] Smieszek T., Pouwels K.B., Dolk F.C.K., Smith D.R., Hopkins S., Sharland M., Hay A.D., Moore M.V., Robotham J.V. (2018). Potential for reducing inappropriate antibiotic prescribing in English primary care. J. Antimicrob. Chemother..

[6] European Centre for Disease Prevention and Control (2017). Summary of the Latest Data on Antibiotic Consumption in EU: 2017.

[7] Pouwels K.B., Dolk F.C.K., Smith D.R.M., Smieszek T., Robotham J.V. (2018). Explaining variation in antibiotic prescribing between general practices in the UK. J. Antimicrob. Chemother..

[8] Butler C.C., Hood K., Verheij T., Little P., Melbye H., Nuttall J., Kelly M.J., Molstad S., Godycki-Cwirko M., Almirall J. (2009). Variation in antibiotic prescribing and its impact on recovery in patients with acute cough in primary care: Prospective study in 13 countries. BMJ.

[9] Pinder R.J., Berry D., Sallis A., Chadborn T. (2015). Antibiotic Prescribing and Behaviour Change in Healthcare Settings: Literature Review and Behavioural Analysis.

[10] Atkins L.C., Tim, Bondaronek P., Ashiru-Oredope D., Beech E., Herd N., Lyon V., González-Iraizoz M., Hopkins S., McNulty C. Which behaviours and mechanisms of action are targeted by national antimicrobial stewardship interventions for patients, community pharmacy staff, primary care prescribers, providers and commissioners and what is their content?.

[11] Borek A.J., Nia W.M.R., Atkins L., Sallis A., Tonkin-Crine S. (2019). Exploring the Implementation of Interventions to Reduce Antibiotic Use (ENACT Study): Report.

[12] Tonkin-Crine S., Yardley L., Little P. (2011). Antibiotic prescribing for acute respiratory tract infections in primary care: A systematic review and meta-ethnography. J. Antimicrob. Chemother..

[13] Germeni E., Frost J., Garside R., Rogers M., Valderas J.M., Britten N. (2018). Antibiotic prescribing for acute respiratory tract infections in primary care: An updated and expanded meta-ethnography. Br J. Gen. Pr..

[14] Tonkin-Crine S.K., San Tan P., van Hecke O., Wang K., Roberts N.W., McCullough A., Hansen M.P., Butler C.C., Del Mar C.B. (2017). Clinician-targeted interventions to influence antibiotic prescribing behaviour for acute respiratory infections in primary care: An overview of systematic reviews. Cochrane Database Syst. Rev..

[15] Köchling A., Löffler C., Reinsch S., Hornung A., Böhmer F., Altiner A., Chenot J.F. (2018). Reduction of antibiotic prescriptions for acute respiratory tract infections in primary care: A systematic review. Implement. Sci..

[16] McDonagh M.S., Peterson K., Winthrop K., Cantor A., Lazur B.H., Buckley D.I. (2018). Interventions to reduce inappropriate prescribing of antibiotics for acute respiratory tract infections: Summary and update of a systematic review. J. Int. Med. Res..

[17] National Health Service (2019). The NHS Long Term Plan.

[18] Michie S., Johnston M., Francis J., Hardeman W., Eccles M. (2008). From theory to intervention: Mapping theoretically derived behavioural determinants to behaviour change techniques. Appl. Psychol..

[19] Yardley L., Morrison L., Bradbury K., Muller I. (2015). The person-based approach to intervention development: Application to digital health-related behavior change interventions. J. Med. Internet Res..

[20] Gulliford M.C., van Staa T., Dregan A., McDermott L., McCann G., Ashworth M., Charlton J., Little P., Moore M.V., Yardley L. (2014). Electronic health records for intervention research: A cluster randomized trial to reduce antibiotic prescribing in primary care (eCRT study). Ann. Fam. Med..

[21] Little P., Stuart B., Francis N., Douglas E., Tonkin-Crine S., Anthierens S., Cals J.W., Melbye H., Santer M., Moore M. (2013). Effects of internet-based training on antibiotic prescribing rates for acute respiratory-tract infections: A multinational, cluster, randomised, factorial, controlled trial. Lancet.

[22] Francis N.A., Butler C.C., Hood K., Simpson S., Wood F., Nuttall J. (2009). Effect of using an interactive booklet about childhood respiratory tract infections in primary care consultations on reconsulting and antibiotic prescribing: A cluster randomised controlled trial. BMJ.

[23] Cox C.M., Jones M. (2001). Is it possible to decrease antibiotic prescribing in primary care? An analysis of outcomes in the management of patients with sore throats. Fam. Pract..

[24] McNulty C.A., Kane A., Foy C.J., Sykes J., Saunders P., Cartwright K.A. (2000). Primary care workshops can reduce and rationalize antibiotic prescribing. J. Antimicrob. Chemother..

[25] Butler C.C., Simpson S.A., Dunstan F., Rollnick S., Cohen D., Gillespie D., Evans M.R., Alam M.F., Bekkers M.J., Evans J. (2012). Effectiveness of multifaceted educational programme to reduce antibiotic dispensing in primary care: Practice based randomised controlled trial. BMJ.

[26] Little P., Hobbs F.R., Moore M., Mant D., Williamson I., McNulty C., Cheng Y.E., Leydon G., McManus R., Kelly J. (2013). Clinical score and rapid antigen detection test to guide antibiotic use for sore throats: Randomised controlled trial of PRISM (primary care streptococcal management). BMJ.

[27] McNulty C., Hawking M., Lecky D., Jones L., Owens R., Charlett A., Butler C., Moore P., Francis N. (2018). Effects of primary care antimicrobial stewardship outreach on antibiotic use by general practice staff: Pragmatic randomized controlled trial of the TARGET antibiotics workshop. J. Antimicrob. Chemother..

[28] Hallsworth M., Chadborn T., Sallis A., Sanders M., Berry D., Greaves F., Clements L., Davies S.C. (2016). Provision of social norm feedback to high prescribers of antibiotics in general practice: A pragmatic national randomised controlled trial. Lancet.

[29] TARGET (Treat Antibiotics Responsibly, Guidance, Education, Tools) Antibiotic Toolkit. https://www.rcgp.org.uk/clinical-and-research/resources/toolkits/target-antibiotic-toolkit.aspx.

[30] STAR: Stemming the Tide of Antibiotic Resistance. https://www.healthcarecpd.com/course/star-stemming-the-tide-of-antibiotic-resistance.

[31] Alison R.L.D.M., Beech E., Ashiru-Oredope D., Costelloe C., Owens R., McNulty C.A.M. What resources do NHS commissioning organisations use to support antimicrobial stewardship in primary care in England?.

[32] Jones L.F., Hawking M.K., Owens R., Lecky D., Francis N.A., Butler C., Gal M., McNulty C.A.M. (2017). An evaluation of the TARGET (Treat Antibiotics Responsibly; Guidance, Education, Tools) Antibiotics Toolkit to improve antimicrobial stewardship in primary care—Is it fit for purpose?. Fam. Pract..

[33] Greaves C., Sheppard K.E., Abraham C., Hardeman W., Roden M., Evans P.H., Schwarz P. (2011). Systematic review of reviews of intervention components associated with increased effectiveness in dietary and physical activity interventions. BMC Public Health.

[34] Michie S., Abraham C., Whittington C., McAteer J., Gupta S. (2009). Effective techniques in healthy eating and physical activity interventions: A meta-regression. Health Psychol..

[35] Cooke J., Butler C., Hopstaken R., Dryden M.S., McNulty C., Hurding S., Moore M., Livermore D.M. (2015). Narrative review of primary care point-of-care testing (POCT) and antibacterial use in respiratory tract infection (RTI). BMJ Open Respir. Res..

[36] Little P., Stuart B., Francis N., Douglas E., Tonkin-Crine S., Anthierens S., Cals J.W., Melbye H., Santer M., Moore M. (2019). Antibiotic Prescribing for Acute Respiratory Tract Infections 12 Months After Communication and CRP Training: A Randomized Trial. Ann. Fam. Med..

[37] Huddy J.R., Ni M.Z., Barlow J., Majeed A., Hanna G.B. (2016). Point-of-care C reactive protein for the diagnosis of lower respiratory tract infection in NHS primary care: A qualitative study of barriers and facilitators to adoption. BMJ Open.

[38] Eley C.V., Sharma A., Lecky D.M., Lee H., McNulty C.A.M. (2018). Qualitative study to explore the views of general practice staff on the use of point-of-care C reactive protein testing for the management of lower respiratory tract infections in routine general practice in England. BMJ Open.

[39] Michie S., Atkins L., West R. (2014). The behaviour change wheel. A Guide to Designing Interventions.

