# Supplementary material for: How Can National Antimicrobial Stewardship Interventions in Primary Care Be Improved? A Stakeholder Consultation
Source: Antibiotics (Basel). 2019 Oct 31;8(4):207. doi: 10.3390/antibiotics8040207 (PMC6963414; doi:10.3390/antibiotics8040207)
Supplement: Supplementary file 1 [file antibiotics-08-00207-s001.pdf]

antibiotics

MDPI

Supplementary Materials and Data for the Article:

Borek, A.J.; Wanat, M.; Sallis, A.; Ashiru-Oredope, D.; Atkins, L.; Beech, E.; Hopkins, S.; Jones, L.; McNulty, C.; Shaw, K.; Taborn, E.; Butler, C.; Chadborn, T.; Tonkin-Crine S. How can national antimicrobial stewardship interventions in primary care be improved? A stakeholder consultation. *Antibiotics*, *8,* 207. Doi: 10.3390/antibiotics8040207

Contents

Box S1. Summary findings from stakeholder consultation: relevant to all settings 2

Box S2. Summary findings from stakeholder consultation: relevant to general practice 3

Box S3. Summary findings from stakeholder consultation: relevant to out-of-hours........... 4

Box S4. Summary findings from stakeholder consultation: relevant to community pharmacy 4

Table S1. All identified suggestions for interventions... 1

Table S2. Stakeholder ratings of interventions for general practice 13

Table S3. Stakeholder ratings of interventions for out-of-hours 17

Table S4. Stakeholder ratings of interventions for walk-in/urgent-care centers 21

Table S5. Stakeholder ratings of interventions for community pharmacy 23

Box S1. Summary findings from stakeholder consultation: relevant to all settings

Facilitating/helping to change antibiotic prescribing and implement interventions:

- Availability of many good interventions.
- Sufficient basic knowledge and awareness of guidelines (i.e., everyone knows what they should be doing).
- Consistency of messages and advice given across settings (pharmacies, general practices, OOH, hospitals etc.).
- Seeing a value in an intervention (otherwise it can be viewed as a 'tick box exercise').
- Prioritizing AMS in everything healthcare professionals do, rather than seeing it as a separate issue.
- Credibility and respectability of people/organizations promoting and providing AMS training and resources (e.g., TARGET toolkit being underwritten by the Royal College of General Practitioners).
- Feedback on individual prescribing rates.
- Feedback on local resistance patterns.
- Online training, especially if it is mandatory and people see value in it (otherwise there is no time to complete it).
- Easy access to patient leaflets (e.g., incorporated into clinical systems).
- Case studies to see how point-of-care CRP tests can work.

Barriers to changing antibiotic prescribing and implementing interventions:

- Differences between local prescribing guidelines causing confusion about appropriate prescribing.
- Guideline 'overload' and lack of time to read prescribing guidelines.
- As many AMS interventions are available and can be accessed in different places/ways, it is unclear which interventions should be used.
- Individual personalities and attitudes to managing risk in healthcare (i.e., low tolerance of risk can increase antibiotic prescribing).
- Difficulty addressing inappropriate prescribing of more senior prescribers by more junior/inexperienced professionals.
- Limited access to healthcare services increasing likelihood of prescribing 'just in case' (e.g., Friday afternoon prescribing).
- Decision-making fatigue linked to workload and time when the prescribing decisions are made.
- Insufficient connectedness and work between networks and organizations ('working in silos'; e.g., commissioners tend to communicate with GPs but not (so much) with OOH and pharmacies).
- Prescribers (e.g., in OOH, locums, dentists) not using their unique prescriber codes, limiting monitoring and accountability for prescribing.
- Insufficient understanding of the importance and consequences of AMR.
- Patient expectations for antibiotics, which may be fueled by other healthcare professionals (e.g., community pharmacist or nurse) or past prescribing. The stakeholders noted that this is slowly improving with due to national campaigns.
- Concern about risks of not prescribing and sepsis.

Suggestions for intervention improvements or new interventions:

- Offering financial incentives for intended change, e.g., for completing AMS training and adherence to guidelines (e.g., as part of professional development, appraisal, contracts).
- Making AMS training mandatary (e.g., as part of clinical professional development or appraisal).

- Tailoring education / training to patient-facing and not patient-facing roles.
- Identifying high prescribers and providing interventions and support tailored to them.
- Providing point-of-care diagnostics, such as CRP tests. (Although the stakeholders had ambivalent views whether they would help or not.)
- Making professional networks more inclusive and multi-professional. For example, including pharmacists and OOH staff in primary care networks when disseminating information or providing AMS training; inviting practice pharmacists to CCG / locality meetings so that they are aware of the local AMS agendas and actions.
- Organizing multi-professional small group-based learning sets.
- Making the TARGET toolkit into an app.
- Providing (better) access to data on local antimicrobial resistance. Linking antibiotic prescribing data with AMR data.
- Addressing concerns about sepsis (e.g., a checklist of red-flag symptoms targeted at general practice, OOH or pharmacy (currently such information is targeted at hospitals)).
- Incorporating tools / interventions into the clinical systems nationally to make them easy to use (e.g., links to guidelines, clinical scores, leaflets, etc.).

Box S2. Summary findings from stakeholder consultation: relevant to general practice

Facilitating/helping to change antibiotic prescribing and implement interventions:

- Offering financial incentives with targets.
- Point-of-care CRP tests used to confirm diagnoses and educate patients. (Mixed views.)

Barriers to changing antibiotic prescribing and implementing interventions:

- Hospital consultants asking GPs to prescribe antibiotics, even if not necessary.
- Timing of consultations and limited access to healthcare (e.g., Friday afternoon prescribing).
- Time pressure and workload (prescribing seen as quicker).
- Decision-making fatigue.
- Recording signs and symptoms as indicating the need for antibiotics if a prescriber wants to prescribe (eve if it may not be appropriate/necessary).
- Prescribers (e.g., locums) using other GPs' codes so it is unclear who issued prescriptions.
- Cost, doubts about accuracy and unintended consequences as barriers/disadvantaged of point-of-care CRP testing.

Suggestions for intervention improvements or new interventions:

- Audit of prescribing in all practices; providing feedback and identifying and addressing the underlying reasons for high prescribing.
- Audit of individual prescribing; providing feedback and working with individual high prescribers to address reasons for high/inappropriate prescribing.
- Making audit of prescribing in practices mandatory.
- Providing training to the 'front-of-the-house' practice staff (including receptionists and community pharmacy assistants) in giving self-care advice and signposting patients.
- Peer review of prescribing between GPs (e.g., as part of an appraisal process).
- Incorporate guidelines into clinical system and decision support tools.

Box S3. Summary findings from stakeholder consultation: relevant to out-of-hours

In addition to similar influences on prescribing as in general practice (as above), the following were identified as specific to OOH:

Facilitating/helping to change antibiotic prescribing and implement interventions:

- Manual audit of individual prescribing decisions and specific feedback to prescribers (e.g., links to guidelines).

Barriers to changing antibiotic prescribing and implementing interventions:

- If the patient is re-consulting and the 'story' they present (e.g., how many times were already seen by a healthcare professional).
- Lack of awareness of local guidelines as in OOH there are often prescribers from various places and their inductions are very quick.
- Lack of communication from the CCG (e.g., about guidelines updates, training opportunities).
- Audit of prescribing in OOH is difficult as OOH providers don't have specific geographical areas or population.
- Not using unique prescriber codes, making automated prescribing audits not possible.
- Lack of accountability for prescribing as prescribing is not monitored by the CCG.
- Lack of incentives or training offered to OOH (such as those offered to general practices).
- Lack of follow-up of patients to see if the treatment worked stifling learning and confidence in decision making.
- Difficult access to patients' notes due to different computer systems.
- Lack of access to lab results.
- Lack of accountability for using broad-spectrum antibiotics.

Suggestions for intervention improvements or new interventions:

- Developing or improving tools/system/software to audit prescribing and give personalized advice (e.g., pointing out to specific guidelines).
- Audit of individual prescribing to identify inappropriate prescribing and provide personalized advice and/or training.
- Improving dissemination of prescribing guidelines and guideline updates to OOH providers and prescribers.
- Making AMS training (e.g., provided by CCGs, NHS) available to OOH staff.
- Improving dissemination of information about AMS training opportunities to OOH.
- Improving induction of new prescribers in OOH.

Box S4. Summary findings from stakeholder consultation: relevant to community pharmacy

Barriers to changing antibiotic prescribing and implementing AMS interventions:

- Lack of access to point-of-care diagnostics to check whether illness is minor or serious.
- Differences in which services are commissioned by the NHS to those offered in private pharmacies (e.g., point-of-care diagnostics, such as throat swabs offered to patients for a fee in private pharmacies but not commissioned by the NHS).
- Concern about the use of point-of-care diagnostics increasing prescribing for financial benefit.
- Limited influence of the NHS/PHE over private services.
- Many different providers and computer systems that are not always compatible.
- Lack of incentives to change, e.g., to use patient leaflets.

- Low use of patient records in pharmacies (unclear why); easier to access patient records if the pharmacy is on the practice premises and they have a practice computer with access to patient records which helps with collaborative working.

Influences on antibiotic prescribing (may be barriers or facilitators):

- Availability of professionals with varying degrees of training and experience in pharmacy teams (e.g., pharmacist prescribers, dispensers, assistants, pharmacy technicians).
- Degree of confidence and skills of pharmacy staff in asking the right questions and making a decision about what to advise the patient (e.g., to self-care, see a GP or go to a hospital).
- Confidence to give self-care advice is often low leading to over safety-netting and telling too many patients to see a GP.
- How patients present their 'story' and their expectations of treatment:
    - How patients describe their symptoms influences the perception of illness and what to advise.
    - Patients may know who in the pharmacy can prescribe and ask them specifically for antibiotics.
    - Patients may expect free-of-charge medicines.
    - Community pharmacists advising patients to see a GP creates a patient expectation for antibiotics (then harder for GPs to say no).

Suggestions for intervention improvements or new interventions:

- Providing training in giving self-care advice to the whole pharmacy teams in order to:
    - ensure a good level and mix of skills within pharmacy teams (considering varying roles and levels of experience/training);
    - develop confidence to give self-care advice and not automatically prescribe antibiotics or direct patients to a GP;
    - ensure consistent messages about self-care and antibiotics;
    - manage patient expectations for antibiotics.
- Providing training to pharmacy staff that includes:
    - structured way(s) of asking patients the right questions and identifying red-flags;
    - support/encouragement to pharmacy staff to provide self-care advice;
    - consistent ways of providing self-care advice;
    - promoting the use of patient leaflets on symptom duration;
    - promoting the signposting patients to self-help advice online.
- Promoting the use of patient records to review whether antibiotics were prescribed appropriately and encouraging pharmacists to identify and challenge inappropriate decisions.
- Providing access to point-of-care diagnostics to address the lack of confidence in identifying whether illness is minor or serious; this would need to be used together in training on how to use the tests and guidelines.

antibiotics

MDPI

Table S1. All identified suggestions for interventions

Barriers (B) / facilitators (F) addressed[1]	Source of the suggestions	Suggested interventions (intervention modifications or new components)	Included in the survey / excluded with reasons
**RELEVANT TO ALL SETTINGS**			
F: Learning from peers on whether they can improve and how and about alternative prescribing techniques	Stakeholders	1. Multi-disciplinary small group learning (e.g. including GPs, nurses, pharmacists, CCG staff) to identify ways to improve implementation of AMS initiatives and share examples of good practice and actions taken by others as part of AMS.	Included
B: Clinical uncertainty; fear of missing a serious illness as a threat to professional expertise; lack of access to POC diagnostic testing ; F: access to POC diagnostics testing	Stakeholders; research evidence	2. Provide diagnostic CRP testing, including training in using it and interpreting the results.	Included
F: Knowledge of and access to case studies on how CRP testing works*	Stakeholders	3. Provide case studies on implementation of CRP testing to better understand how CRP testing can work in different settings and to provide role models.	Excluded: Not specific enough; healthcare organizations operate differently and it would be difficult to transfer learning; implementation studies are currently underway to provide some examples of implementation.

B: Disintegrated services & communication structures*; F: multi-disciplinary networks*	Stakeholders	4. Co-organize AMS events together with different professional networks (e.g. GPs, nurses, pharmacists, OOH staff) to facilitate multi-disciplinary work and improve dissemination of information about AMS events and training to all professional networks.	Included
F: Advice from / influence of relevant experts, providing / underwriting the intervention by relevant people / organizations, e.g. RCGP, BMA*	Stakeholders; research evidence	5. Use relevant and credible sources to promote AMS and engagement with under-used AMS interventions.	Included
F: Ability to educate patients in consultations; access to and use of patient leaflets helping to explain no-prescribing decisions & providing advice	Stakeholders, research evidence	6. Promote higher uptake and interactive use of patient leaflets (e.g. through training, role models, credible sources and peers promoting them) by all healthcare professionals in general practice, OOH and community pharmacy.	Excluded: Included in the community pharmacy setting only; patient leaflets are already being used in general practice and OOH settings.
B: Inconsistent approaches to antibiotic prescribing; F: adopting guidelines or evidence as a standard practice (with intention to follow them)	Researchers, research evidence	7. Agree on a consistent approach to antibiotic prescribing within an organization (e.g. make an action plan, agree a practice protocol on treating certain infections).	Included
F: Advice from and influence of relevant experts	Researchers	8. Increase and standardize the role of CCG medicines optimization teams and their responsibilities across all CCGs (e.g. all CCGs to have adequate number of prescribing advisors and/or pharmacists to work more closely with practices, OOH and	Included

		community pharmacies (e.g. in auditing prescribing, disseminating information, providing training and advice).	
B: Variation in the skills and experience among staff (in particular reported for pharmacy teams)*	Stakeholders	9. Provide AMS-related training to all patient-facing staff within an organization to improve and minimize variation in skills and to ensure a consistent approach to providing advice to patients and antibiotic prescribing.	Included
F: Ability to elicit and address patient's concerns and expectations; ability to prepare a patient for, and justify, a decision whether to prescribe or not; ability to educate patients in consultations; B: lack of such abilities	Stakeholders; research evidence	10. Promote higher uptake of training (e.g. STAR) including evidence to prescribers that patients expect reassurance and addressing their concerns, not necessarily a prescription, and development of skills, including:<br>- eliciting and addressing patients' concerns & expectations,<br>- preparing patients for a decision whether to prescribe antibiotics or not,<br>- educating patients about self-limiting illness and antibiotics,<br>- providing self-care advice & sign-posting patients to sources of self-care information,<br>- maintaining good relationship with patient and patient satisfaction (in ways other than by giving a prescription when inappropriate),<br>- ability to do that in a time-efficient way.	Excluded:<br>Overlap with existing online trainings; not specific enough in terms of delivery.
B: Poor dissemination of guidelines (in particular to OOH)*	Stakeholders; research evidence	11. Improve dissemination and implementation of (new) guidelines (e.g. CCGs / NHS England improve dissemination of	Excluded:<br>Not specific enough, especially in terms of how

		information, including to OOH providers, offer update training and meetings).	dissemination can be improved (CCG already disseminate guidelines so it remains unclear how else it can be improved.)
B: Lack of communication from CCGs to OOH about AMS*; Lack of training (or invitations to training) on AMS offered to OOH by CCGs*	Stakeholders	12. Improve dissemination and uptake of AMS-related training opportunities, in particular to OOH providers (together with opening existing AMS training to OOH providers).	Excluded: Similar to other suggestions; not specific enough, especially in terms of how dissemination and uptake can be improved.
**RELEVANT TO GENERAL PRACTICE**			
F: Advice from colleagues when uncertain or to reinforce appropriate prescribing decisions, perceptions of own prescribing compared to others	Stakeholders	1. Prescribing advisors or practice prescribing leads to encourage GPs to peer review each other's antibiotic prescribing, review uncertain cases regularly and get advice from peers on what could/should have been done (e.g. as part of an appraisal process or a task within a practice meeting).	Included
F: Having prescribing monitored and audited, receiving feedback on prescribing; B: lack of accountability for prescribing	Stakeholders; research evidence	2. Audit prescribing in all practices, to be done by CCG prescribing advisors or practice prescribing leads, and identify underlying reasons for high/inappropriate prescribing, provide tailored advice to practice prescribers and agree practice action plans.	Included
F: Having prescribing monitored and audited, receiving feedback on	Stakeholders	3. Audit prescribing of individual prescribers in practices, to be done by CCG prescribing advisors or practice prescribing leads,	Included

prescribing; B: lack of accountability for prescribing**;** individual personalities and attitudes to managing risk in healthcare*		provide individual feedback on prescribing, identify individual high prescribers (or those not following guidelines), identify underlying reasons for high/inappropriate prescribing, provide tailored advice and agree individual action plans.	
B: Lack of consistency in auditing prescribing*	Stakeholders	4. Make prescribing audit mandatory in all practices nationally (e.g. as part of CQC or other CCG or national targets for practices).	Excluded: Seen as unrealistic; audit is already part of the CQC.
B: (Belief about) limited access to GP / medical services lowering threshold for prescribing	Stakeholders	5. Provide information on the exact (amount of) time that there is no access to healthcare available locally to patients and encourage all prescribers to know the exact times and locations of available healthcare services to provide to patients (e.g. as a leaflet).	Included
F: Awareness/knowledge of evidence & guidelines; easy access to patient leaflets*; B: lack of time to engage with interventions*; clinical uncertainty	Stakeholders	6. Clinical system providers to incorporate interventions (e.g. guidelines, leaflets, clinical decision support tools) into all clinical systems nationally.	Included
F: Use of financial incentives to change antibiotic prescribing; B: lack of incentives to change or engage with interventions*	Stakeholders	7. All CCGs to provide financial incentives to general practices for intended behaviors (e.g. to engage with interventions, adhere to guidelines, do prescribing audits) or outcomes (e.g. reduce antibiotic prescribing).	Excluded: Questioned the evidence on whether financial incentives facilitate sustainable change; long-term financial incentives seen as unrealistic; some

			have already been implemented.
B: Lack of incentives to engage with interventions*	Stakeholders	8. Make AMS training mandatory (e.g. evidence-based STAR training as part of CQC, contracts, targets) if practice prescribing rates reach a certain threshold (e.g. top 25% nationally or locally).	Included
B: Lack of understanding of and awareness of AMR*; doubt about impact of GPs' prescribing on AMR; F: belief that GPs' prescribing meaningfully contributes to AMR	Stakeholders; research evidence	9. Improve dissemination and awareness of data on local antimicrobial resistance patterns and evidence that links it with prescribing rates, describing the relation between antibiotic prescribing and antimicrobial resistance (e.g. by CCG prescribing advisors communicating this information to practices, together with feedback on antibiotic prescribing rates as part of regular reviews, audits or training).	Included
B: Prescribers not using unique codes making it difficult to automate prescribing audit*	Stakeholders	10. Promote or regulate the use of unique prescriber codes in order to be able to provide individual prescribing feedback.	Included
F: Ability to preserve a good relationship and patient satisfaction in other ways than prescribing	Researchers	11. Improve dissemination of evidence to general practice prescribers (by peers or other credible sources) that patients most often want reassurance, and addressing their concerns in consultations is sufficient, without necessarily giving an antibiotic prescription (e.g. as part of existing training delivered to prescribers).	Excluded: Already part of existing, nationally implemented online STAR training.
B: Responding to immediate pressures (e.g. patient in front) over long-term consequences of AMR; wanting to prevent re-consultations	Research evidence	12. Emphasize the benefits to prescribers of not prescribing when inappropriate and of taking time to educate patients and provide self-care advice (e.g. lower re-consultations, future consultations and patient expectation of antibiotics.	Excluded: Already available as part of the TARGET resources.

by giving a prescription; F: Wanting to save time and prevent future consultations by investing time in educating patients about self-care of self-limiting illnesses			
(no specific barriers / facilitators identified in literature or by stakeholders)	Research evidence	13. Implement computer prompts into clinical systems nationally and provide training in how to use the prompts to optimize antibiotic prescribing (e.g. reminders to reduce broad-spectrum antibiotics, to use delayed prescriptions, patient leaflets).	Excluded:<br>Already being developed as part of the TARGET resources; lacking specificity on what prompts would need to be implemented. Incorporated as part of suggestion #7 for general practice (i.e. incorporating interventions into clinical systems).
(no specific barriers / facilitators identified in literature or by stakeholders)	Research evidence	14. Appoint AMS lead GPs in all practices to lead on AMS-related issues, for example, by organizing practice meetings about AMS, disseminating information about new guidelines, encouraging peers to implement interventions.	Included
(no specific barriers / facilitators identified in literature or by stakeholders)	Research evidence	15. CCG prescribing advisors to provide encouragement and verbal persuasion to promote AMS-related behavior change (e.g.	Excluded:

		encouraging /persuading GP directly in what they could do to reduce antibiotic prescribing).	Already part of an existing TARGET intervention/ resources.
**RELEVANT TO OUT-OF-HOURS**			
F: Having prescribing monitored and audited, receiving feedback on prescribing; B: lack of accountability for prescribing; auditing and benchmarking prescribing in OOH impossible or difficult due to not being linked to population or area*; B: Lack of understanding of & accountability for prescribing broad-spectrum antibiotics in OOH*	Stakeholders	1. Manual audit of (sample of) individual prescriptions (prescribing decisions) in OOH (especially the use of broad-spectrum antibiotics), identify underlying reasons for high/inappropriate prescribing, followed by provision of personalized feedback and advice/training to prescribers, and agreed action plans.	Excluded: Software exist that does it; developing tools / system to enable audit is already included in another suggestion.
B: Auditing and benchmarking prescribing in OOH impossible or difficult due to not being linked to population or area*	Stakeholders	2. Develop tools/system to enable audit of prescribing in OOH and provision of personalized feedback and advice.	Included
B: Lack of consistency in auditing prescribing*	Stakeholders	3. Make prescribing audit in OOH mandatory.	Included
B: Lack of awareness/ knowledge of local guidelines by new/locum GPs in OOH*	Stakeholders	4. Improve induction for new prescribers in OOH to ensure knowledge of local AMS-relevant guidelines, specifically indications for antibiotic prescribing, information on first-line antibiotics, practice agreed action plans / approaches to prescribing antibiotics.	Included

B: (Belief about) limited access to GP / medical services lowering threshold for prescribing	Stakeholders	5. Provide information on the exact (amount of) time that there is no access to healthcare available locally to patients and encourage all prescribers to know the exact times and locations of available healthcare services to provide to patients (e.g. as a leaflet).	Excluded: Not relevant to OOH. Included in general practice setting.
F: Awareness/knowledge of evidence & guidelines; easy access to patient leaflets*; B: lack of time to engage with interventions*; clinical uncertainty	Stakeholders	6. Clinical system providers to incorporate interventions (e.g. guidelines, leaflets, clinical decision support tools) into all clinical systems nationally.	Included
B: Lack of understanding of and awareness of AMR*; doubt about impact of GPs' prescribing on AMR; F: belief that GPs' prescribing meaningfully contributes to AMR	Stakeholders, research evidence	7. Improve dissemination and awareness of data on local antimicrobial resistance patterns and evidence that links it with prescribing rates describing the relation between antibiotic prescribing and antimicrobial resistance (e.g. by CCG prescribing advisors communicating this information to OOH, together with feedback on antibiotic prescribing rates as part of regular reviews, audits or training).	Included
B: Prescribers not using unique codes making it difficult to automate prescribing audit*	Stakeholders	8. Promote or regulate the use of unique prescriber codes in order to be able to provide individual prescribing feedback.	Included
F: Belief that delayed prescriptions can be helpful; B: Belief that delayed prescribing is not an effective strategy for appropriate/ prudent antibiotic prescribing; perception that patients	Research evidence	9. Promote increased use of delayed prescriptions (instead of immediate prescriptions) by providing evidence to prescribers on why delayed prescribing is beneficial to them and their patients and on how patients typically use delayed prescriptions.	Included

use delayed prescriptions inappropriately (lack of feedback on how patients use delayed prescriptions)			
F: Ability to preserve a good relationship and patient satisfaction in other ways than prescribing	Researchers	10. Improve dissemination of evidence to prescribers in OOH (by peers or other credible sources) that patients most often want reassurance, and addressing their concerns in consultations is sufficient, not necessarily a prescription (e.g. as part of existing training delivered to prescribers).	Excluded:<br>Important to all settings and, to some extent, already happening; unspecific in terms of how to improve dissemination.
B: Responding to immediate pressures (e.g. patient in front) over long-term consequences of AMR; wanting to prevent re-consultations by giving a prescription; F: Wanting to save time and prevent future consultations by investing time in educating patients about self-care of self-limiting illnesses	Research evidence	11. Emphasize the benefits to prescribers of not prescribing when inappropriate and of taking time to educate patients and provide self-care advice (e.g. lower re-consultations, future consultations and patient expectation of antibiotics).	Excluded:<br>Already available as part of the TARGET resources.
(no specific barriers / facilitators identified in literature or by stakeholders)	Research evidence	12. Appoint AMS lead GPs in all OOH to lead on AMS-related issues, for example, by organizing practice meetings about AMS, disseminating information about new guidelines, encouraging peers to implement interventions.	Included

(no specific barriers / facilitators identified in literature or by stakeholders)	Research evidence	13. CCG prescribing advisors to provide encouragement and verbal persuasion to promote AMS-related behavior change (e.g. encouraging /persuading GP directly in what they could do to reduce antibiotic prescribing).	Excluded: Already covered by a suggestion for all settings (i.e. use respected and trusted, national and local experts… to promote AMS…).
B: Lack of follow-up of patients between general practice and OOH	Researchers	14. Make patient information and history available on OOH IT system, and the OOH information available on GP IT system for a GP to be able to follow-up afterwards.	Included
**RELEVANT TO COMMUNITY PHARMACY**			
B: Low use of patient records in community pharmacy to review prescribing*	Stakeholders	1. Promote the use of patient records by pharmacists to review whether antibiotics were prescribed appropriately and encourage pharmacists to flag GP prescribing decisions if antibiotics were prescribed inappropriately (not according to guidelines).	Included (spilt into two suggestions)
B: Lack of skills of community pharmacy staff to ask questions and make decisions what to advise patients*; lack of confidence of community pharmacy staff on what to advise and over-advising to see a GP*; patient’s concern about illness*	Stakeholders	2. Provide training in structured way(s) of asking patients the right questions and identifying red-flags to help decide what to advise patients (e.g. whether to give them self-help advice or suggest seeing a GP).	Included

B: Low use of patient records in community pharmacy to review prescribing*; lack of review of COPD back up antibiotics (suggested by research team)	Stakeholders, researchers	3. Pharmacy staff to prompt GPs to review long-term and repeat antibiotic prescriptions (e.g. for COPD patients).	Included
F: Access to and use of patient leaflets (helping to explain no-antibiotic prescribing decisions & providing advice)	Stakeholders (suggestion wasn't specific to setting), researchers (narrowed down to this setting as leaflets already available in GP, OOH settings), research evidence	4. Promote routine interactive use of patient leaflets with patients with self-limiting infections (e.g. through training, role models, respected and trusted experts promoting use of leaflets).	Included

Note:

[1] Barriers and facilitators were derived from, and defined on the basis of, a qualitative literature review (reported in: Borek et al. *Exploring the implementation of interventions to reduce antibiotic use (ENACT study): report. 2019. Public Health England)* and may not match exactly with the intervention suggestions. Barriers suggested by the stakeholders are indicated with an asterisk (*). Barriers and facilitators for community pharmacy were only reported by stakeholders due to lack of research studies identified in this setting.

Abbreviations used in the table: AMR – antimicrobial stewardship, B – barrier, F – facilitator, GP – general practitioner, OOH – out of hours.

Table S2. Stakeholder ratings of interventions for general practice

**Intervention** (in order of percentage APEASE score)	**Number of respondents considering the intervention…**							**APEASE score**	
	**Relevant to this setting**	**A** Affordable	**P** Practical	**E** Effective	**A** Acceptable	**S** Safe	**E** Equitable	**Total**	**% maximum score**
Prescribing advisors or practice prescribing/AMS leads to carry out standardized quality improvement (e.g. supported by IT system functionality) and use prescribing data to identify underlying reasons for high / inappropriate antibiotic prescribing, provide tailored advice to prescribers and agree practice action plans (e.g. practice plan to reduce immediate antibiotic prescribing for acute cough).	11/11	8	8	10	10	10	10	56/66	84.9%
Multi-disciplinary small group learning (e.g. including local GPs, nurses, pharmacists, CCG staff) to identify ways to improve implementation of antimicrobial stewardship (AMS) initiatives and share local examples of good practice and actions taken by others as part of AMS.	13/14	10	11	13	12	13	12	71/84	84.5%
Appoint AMS lead GPs in all practices to lead on AMS-related issues, e.g., by organizing practice meetings about AMS, disseminating information about new guidelines, encouraging peers to implement interventions.	11/11	9	8	9	8	11	10	55/66	83.3%

Audit prescribing of individual prescribers in general practices, to be done by local (CCG) prescribing advisors, practice prescribing/AMS leads or practice pharmacists, and provide individual feedback on prescribing, identify underlying reasons for high / inappropriate antibiotic prescribing, provide tailored advice and agree individual action plans (e.g. individual prescriber's plan to reduce immediate antibiotic prescribing for acute cough).	10/11	8	9	10	8	10	10	55/66	83.3%
Promote or regulate the use of unique prescriber codes to be able to provide individual prescribing feedback.	11/11	9	8	9	8	10	9	53/66	80.3%
Clinical system providers to incorporate interventions (e.g. guidelines, leaflets, clinical decision support tools, computer prompts to use delayed/back-up prescriptions, computer prompts to reduce broad-spectrum antibiotics) into all clinical systems nationally and commissioners to ensure that practices activate and are aware of these functions on their clinical system.	10/11	6	9	8	9	10	9	51/66	77.3%
Improve dissemination and awareness of data on local antimicrobial resistance patterns and evidence that links it with prescribing rates, describing the relation between antibiotic prescribing and antimicrobial resistance (e.g. by prescribing	9/11	8	7	6	9	9	9	48/66	72.7%

advisors communicating this information to practices, together with feedback on AMR and antibiotic prescribing rates as part of regular reviews, audits or training).									
Promote increased use of delayed / back-up prescriptions (instead of immediate prescriptions) by providing online skills training to prescribers. Training to include information on why delayed/back-up prescribing is beneficial, how patients use delayed/back-up prescriptions, and by a practice agreeing on a process to provide delayed/back-up prescriptions.	9/11	8	7	8	8	9	8	48/66	72.7%
Use respected and trusted, national and local experts with relevant professional backgrounds to promote AMS and engagement with under-used AMS interventions by giving talks and endorsing AMS approaches.	12/14	7	7	9	12	12	9	56/84	66.7%
Agree on a consistent local approach to antibiotic prescribing within an organisation, such as a general practice, out-of-hours, walk-in centre or community pharmacy, for example, by agreeing an AMS-related action plan, a practice protocol on treating certain infections and/or following national or local guidelines.	10/14	10	9	9	9	10	9	56/84	66.6%

Increase staff time available to work on AMS within commissioning teams and standardise the AMS-related roles; for example, all commissioners to have adequate number of prescribing advisors and/or pharmacists to work more closely with practices, OOH and community pharmacies (e.g. by auditing prescribing, disseminating information, providing training and advice).	11/14	6	7	10	10	11	10	54/84	64.3%
Provide online AMS training to all patient-facing staff within an organization to improve (and minimize variation in) skills to ensure a consistent approach to providing advice to patients and antibiotic prescribing for respiratory tract infections.	11/14	11	8	6	8	11	9	53/84	63.1%
Local prescribing advisors or practice prescribing/AMS leads to encourage GPs to peer review each other's antibiotic prescribing, review uncertain cases regularly and promote discussion on alternative approaches to immediate prescribing (e.g. as a task within a practice meeting).	10/11	7	5	6	6	8	7	39/66	59.1%
Make AMS training mandatory (e.g. evidence-based training as part of CQC, contracts, targets) if practice prescribing rates reach a certain threshold (e.g. top 25% nationally or locally).	9/11	6	6	6	3	8	9	38/66	57.6%

Co-organise national AMS events together with different professional networks (e.g. GPs, nurses, pharmacists, OOH staff) to facilitate multi-disciplinary work and improve dissemination of information about AMS and training to all relevant professional networks.	10/14	5	7	7	8	10	8	45/84	53.6%
Provide diagnostic point-of-care CRP testing, including training in using it, interpreting the results and maintaining the equipment.	11/14	2	4	7	6	11	6	37/84	44.0%
Provide information on opening hours of all local healthcare services for prescribers and patients to know what care is available to patients outside GP hours (e.g. as a leaflet, on a practice website) to prevent higher prescribing on Fridays.	5/11	5	6	2	6	5	5	21/66	31.8%

Table S3. Stakeholder ratings of interventions for out-of-hours

**Intervention**	**Number of respondents considering the intervention…**							**APEASE score**	
	**Relevant to this setting**	**A** Affordable	**P** Practical	**E** Effective	**A** Acceptable	**S** Safe	**E** Equitable	**Total**	**% maximum score**
Appoint AMS lead prescriber in all OOH sites to lead on AMS-related issues, e.g., by organising meetings about AMS, disseminating information about new	6/6	5	5	5	6	6	6	33/36	91.7%

guidelines, encouraging peers to implement interventions.									
Develop tools / system to enable audit of prescribing in OOH and provision of personalised feedback and advice.	5/6	4	4	5	5	5	5	28/36	77.8%
Improve induction for new prescribers in OOH to ensure knowledge of local AMS-relevant guidelines (e.g. indications for antibiotic prescribing, first-line antibiotics) and organisation-agreed approaches to prescribing antibiotics.	5/6	4	4	5	5	5	5	28/36	77.8%
Promote increased use of delayed / back up prescriptions (instead of immediate prescriptions) by providing online skills training to prescribers. Training to include information on why delayed/back-up prescribing is beneficial, how patients use delayed/back up prescriptions, how to discuss delayed/back up prescriptions with patients, and how it can be used in OOH.	5/6	4	4	4	4	5	5	26/36	72.2%
Promote or regulate the use of unique prescriber codes in order to provide individual prescribing feedback.	5/6	4	3	4	4	5	5	25/36	69.4%
Clinical system providers to incorporate interventions (e.g. guidelines, leaflets, clinical decision support tools, computer prompts to use delayed/back-up prescriptions, computer prompts	5/6	3	4	4	4	5	5	25/36	69.4%

to reduce broad-spectrum antibiotics) into all clinical systems nationally and commissioners/providers to ensure these are activated and OOH staff are aware of these functions on their clinical system.									
Make patient information and history available on OOH IT system, and the OOH information available on GP IT system for a GP to be able to follow-up afterwards.	5/6	5	1	4	5	5	5	25/36	69.4%
Multi-disciplinary small group learning (e.g. including local GPs, nurses, pharmacists, CCG staff) to identify ways to improve implementation of antimicrobial stewardship (AMS) initiatives and share local examples of good practice and actions taken by others as part of AMS.	13/14	7	6	10	8	11	12	54/84	64.3%
Provide online AMS training to all patient-facing staff within an organisation to improve (and minimise variation in) skills to ensure a consistent approach to providing advice to patients and antibiotic prescribing for respiratory tract infections.	12/14	12	8	7	8	11	8	54/84	64.3%
Agree on a consistent local approach to antibiotic prescribing within an organisation, such as a general practice, out-of-hours, walk-in centre or community pharmacy, for example, by agreeing an AMS-related	10/14	10	8	9	8	9	10	54/84	64.3%

action plan, a practice protocol on treating certain infections and/or following national or local guidelines.									
Use respected and trusted, national and local experts with relevant professional backgrounds to promote AMS and engagement with under-used AMS interventions by giving talks and endorsing AMS approaches.	10/14	5	7	8	11	12	10	53/84	63.1%
Increase staff time available to work on AMS within commissioning teams and standardise the AMS-related roles; for example, all commissioners to have adequate number of prescribing advisors and/or pharmacists to work more closely with practices, OOH and community pharmacies (e.g. by auditing prescribing, disseminating information, providing training and advice).	10/14	7	7	9	9	10	10	52/84	61.9%
Improve dissemination and awareness of data on local antimicrobial resistance patterns and evidence that links it with prescribing rates, describing the relation between antibiotic prescribing and antimicrobial resistance (e.g. by prescribing advisors communicating this information to OOH, together with feedback on AMR and antibiotic prescribing rates as part of regular reviews, audits or training).	4/6	3	4	3	4	4	4	22/36	61.1%

Make antibiotic prescribing / infection audit in OOH mandatory.	5/6	3	3	2	3	4	4	22/36	61.1%
Provide diagnostic point-of-care CRP testing, including training in using it, interpreting the results and maintaining the equipment.	11/14	4	6	8	5	11	8	42/84	50.0%
Co-organise national AMS events together with different professional networks (e.g. GPs, nurses, pharmacists, OOH staff) to facilitate multi-disciplinary work and improve dissemination of information about AMS and training to all relevant professional networks.	8/14	3	6	6	5	7	7	34/84	40.5%

Table S4. Stakeholder ratings of interventions for walk-in/urgent-care centers

**Intervention**	**Number of respondents considering the intervention…**							**APEASE score**	
	**Relevant to this setting**	**A** Affordable	**P** Practical	**E** Effective	**A** Acceptable	**S** Safe	**E** Equitable	**Total**	**% maximum score**
Agree on a consistent local approach to antibiotic prescribing within an organisation, such as a general practice, out-of-hours, walk-in centre or community pharmacy, for example, by agreeing an AMS-related action plan, a practice protocol on treating certain infections and/or following national or local guidelines.	9/13	9	8	8	8	9	9	51/78	65.4%

Provide online AMS training to all patient-facing staff within an organisation to improve (and minimise variation in) skills to ensure a consistent approach to providing advice to patients and antibiotic prescribing for respiratory tract infections.	11/13	11	7	7	8	9	7	49/78	62.8%
Multi-disciplinary small group learning (e.g. including local GPs, nurses, pharmacists, CCG staff) to identify ways to improve implementation of antimicrobial stewardship (AMS) initiatives and share local examples of good practice and actions taken by others as part of AMS.	12/13	7	6	8	7	10	10	48/78	61.5%
Increase staff time available to work on AMS within commissioning teams and standardise the AMS-related roles; for example, all commissioners to have adequate number of prescribing advisors and/or pharmacists to work more closely with practices, OOH and community pharmacies (e.g. by auditing prescribing, disseminating information, providing training and advice).	9/13	7	7	7	9	9	9	48/78	61.5%
Use respected and trusted, national and local experts with relevant professional backgrounds to promote AMS and engagement with under-used AMS interventions by giving talks and endorsing AMS approaches.	8/13	3	6	7	9	10	8	43/78	55.1%

Provide diagnostic point-of-care CRP testing, including training in using it, interpreting the results and maintaining the equipment.	9/13	3	5	7	6	11	6	38/78	48.7%
Co-organise national AMS events together with different professional networks (e.g. GPs, nurses, pharmacists, OOH staff) to facilitate multi-disciplinary work and improve dissemination of information about AMS and training to all relevant professional networks.	6/13	4	7	4	3	6	6	30/78	38.5%

Table S5. Stakeholder ratings of interventions for community pharmacy

**Intervention**	**Number of respondents considering the intervention…**							**APEASE score**	
	**Relevant to this setting**	**A** Affordable	**P** Practical	**E** Effective	**A** Acceptable	**S** Safe	**E** Equitable	**Total**	**% maximum score**
Provide online AMS training to all patient-facing staff within an organisation to improve (and minimise variation in) skills to ensure a consistent approach to providing advice to patients and antibiotic prescribing for respiratory tract infections.	8/11	8	5	6	6	7	7	39/66	59.1%
Agree on a consistent local approach to antibiotic prescribing within an organisation, such as a general practice, out-of-hours, walk-in centre or	8/11	6	6	6	6	7	8	39/66	59.1%

community pharmacy, for example, by agreeing an AMS-related action plan, a practice protocol on treating certain infections and/or following national or local guidelines.									
Multi-disciplinary small group learning (e.g. including local GPs, nurses, pharmacists, CCG staff) to identify ways to improve implementation of antimicrobial stewardship (AMS) initiatives and share local examples of good practice and actions taken by others as part of AMS.	9/11	6	5	5	5	8	8	37/66	56.1%
Promote routine interactive use of patient leaflets with patients with self-limiting infections (e.g. through training, role models, respected and trusted experts promoting use of leaflets).	2/3	2	2	1	2	2	1	10/18	55.6%
Use respected and trusted, national and local experts with relevant professional backgrounds to promote AMS and engagement with under-used AMS interventions by giving talks and endorsing AMS approaches.	8/11	4	5	6	7	8	6	36/66	54.5%
Increase staff time available to work on AMS within commissioning teams and standardise the AMS-related roles; for example, all commissioners to have adequate number of prescribing advisors and/or pharmacists to work more closely with practices, OOH and community pharmacies (e.g. by	7/11	5	5	5	6	7	7	35/66	53.0%

auditing prescribing, disseminating information, providing training and advice).									
Pharmacy staff to prompt GPs to review long-term and repeat antibiotic prescriptions (e.g. for COPD patients).	2/3	2	1	1	1	2	2	9/18	50.0%
Co-organise national AMS events together with different professional networks (e.g. GPs, nurses, pharmacists, OOH staff) to facilitate multi-disciplinary work and improve dissemination of information about AMS and training to all relevant professional networks.	7/11	3	6	5	4	7	7	32/66	48.5%
Encourage pharmacists to feedback to GPs where antibiotics were not prescribed according to guidelines (e.g. through a checklist for clinical screening, dispensing and handing out antibiotics).	2/3	1	0	2	1	2	2	8/18	44.4%
Promote the use of patient records by pharmacists (e.g. by digital prompts) to review whether antibiotics were prescribed appropriately.	2/3	0	1	1	1	1	2	6/18	33.3%
Provide training and resources to structure the way(s) of asking patients the right questions about self-limiting infections and identifying red flags to help decide what to advise patients (e.g. whether to give self-help advice or suggest seeing a GP).	2/3	1	1	1	1	1	1	6/18	33.3%

Provide diagnostic point-of-care CRP testing, including training in using it, interpreting the results and maintaining the equipment.	5/11	0	2	3	2	5	3	15/66	22.7%

Note to the Tables S2-S5:

The number of respondents that assessed intervention suggestions for different settings varied. Therefore, in the column 'relevant to this setting' the number of respondents who indicated that the suggestion is relevant to that setting is compared to the total number of participants that answered questions in each section (e.g., 5 stakeholders assessed the intervention as relevant to the setting out of 11 who assessed this intervention for this setting according to the APEASE criteria). Most participants assessed interventions in the section for 'all settings' (i.e. 14 for general practice, 14 for OOH, 13 for walk-in centres, and 11 for community pharmacy). Fewer completed the sections specific to each setting (i.e. 11 for general practice, 6 for OOH, and 3 for community pharmacy). The total APEASE scores were compared to the maximum score possible for the number of respondents assessing each intervention for each setting.

© 2019 by the authors. Submitted for possible open access publication under the terms and conditions of the Creative Commons Attribution (CC BY) license (http://creativecommons.org/licenses/by/4.0/).
